# Evaluation of the sedative and physiological effects of xylazine, detomidine, medetomidine and dexmedetomidine in goats

**DOI:** 10.1002/vms3.732

**Published:** 2022-01-11

**Authors:** Atefeh Nahvi, Mohammad Mahdi Molaei, Amir Saeed Samimi, Omid Azari, Hossein Mashayekhi, Fatemeh Ebrahimzadeh

**Affiliations:** ^1^ Department of Clinical Sciences Faculty of Veterinary Medicine Shahid Bahonar University of Kerman Kerman Iran

**Keywords:** α_2_‐adrenergic agonists, goats, physiological variables, sedative effects

## Abstract

**Background:**

Many α_2_‐agonists are commonly used for sedation and analgesia in ruminants.

**Introduction:**

The present study aims to compare the sedative and physiological effects of intravenous (IV) administration of xylazine, detomidine, medetomidine and dexmedetomidine in goats.

**Methods:**

Ten healthy goats aged 6 ± 1 months and weighing 15 ± 2 kg were used in experimental, crossover Latin square, randomised and blinded study. Animals were assigned to five IV treatments: control (normal saline); xylazine (100 μg kg^−1^); detomidine (50 μg kg^−1^); medetomidine (20 μg kg^−1^) and dexmedetomidine (5 μg kg^−1^). The degree of sedation was investigated using a numerical ranking scale of 0–10. Sedation scores were compared at each time using nonparametric (Kruskal–Wallis and Mann–Whitney *U*) tests.

**Results:**

Heart rate (HR), respiratory rate (RR), rectal temperature (RT), ruminal motility and capillary refill time (CRT) were performed before (baseline) and after drug administration. Animals in α_2_‐adrenergic agonist treatments were sedated at 5–60 min. There were no significant differences among α_2_‐adrenergic agonist treatments at 5–60 min in sedation scores. HR significantly decreased from baseline 5–90 min after α_2_‐adrenergic agonists’ administration. Ruminal motility was decreased in α_2_‐adrenergic agonist treatments at 5, 90 and 120 min and absent at 10–60 min. A significant decrease from baseline in RR was detected between 30 and 90 min after α_2_‐adrenergic agonists’ administration. RT was unchanged in any treatment for 120 min. CRT was less than 2 s at all time points following each treatment.

**Conclusions:**

The duration of sedation was up to 60 min after IV administration of xylazine (100 μg kg^−1^), detomidine (50 μg kg^−1^), medetomidine (20 μg kg^−1^) and dexmedetomidine (5 μg kg^−1^) in goats in this study. No significant differences were detected between xylazine, detomidine, medetomidine and dexmedetomidine in goats.

## Abstract 2

The duration of sedation was up to 60 minutes after IV administration of xylazine (100 μg kg^−1^), detomidine (50 μg kg^−1^), medetomidine (20 μg kg^−1^) and dexmedetomidine (5 μg kg^−1^) in goats. No significant differences were detected between xylazine, detomidine, medetomidine and dexmedetomidine in goats.

## 1 INTRODUCTION

In addition to physical restraint, chemical agents are useful and often necessary to ensure immobility and to provide sedation and analgesia for surgical and non‐surgical procedures in the veterinary patients (Kästner, 2006). Many α_2_‐agonists and narcotics are commonly used for sedation, analgesia and anaesthesia in ruminants (Kästner et al., 2007; Kutter et al., 2006).

Since there is a distinct lack of documented information on the sedative effects of α_2_‐adrenergic agonists in goats, the present study aimed to compare the effects of intravenous (IV) administration of xylazine, detomidine, medetomidine and dexmedetomidine on sedation and some physiological variables in goats. The hypothesis of this study is that the sedative and physiological effects would vary between α_2_‐adrenergic agonists in goats.

## 2 MATERIALS AND METHODS

### 2.1 Animals

The study was approved by the animal welfare committee of the Faculty of Veterinary Medicine, Shahid Bahonar University of Kerman (IR.UK.VETMED.REC.1399.014). Ten goats aged 6 ± 1 months and weighing 15 ± 2 kg (mean ± standard deviation) were used. The animals were selected from the Animal Husbandry Unit of Faculty of Veterinary Medicine, Shahid Bahonar University of Kerman (latitude 30^°^19^′^N and longitude 52^°^07^′^E) using a sample lottery method (simple randomisation). All animals were housed under the same environmental, nutritional and management conditions in a same group pen. The animals received constant mixture containing roughages (mainly alfalfa hay and wheat straw) and concentrate (barley grain, soybean meal, wheat bran) based on physiological maintenance during the experiment. The forage/concentrate ratios during the experiment were 90:10. Vitamins and minerals were also provided. Two months before the experiment, animals were treated with broad‐spectrum antiparasitic drugs for probable internal and external parasitic infestation. Health status of all animals was checked routinely by clinical [including heart and respiratory rate (HR and RR), rectal temperature (RT), capillary refill time (CRT) and ruminal motility] and paraclinical examinations. The paraclinical examination consisted of haematological (evaluation of complete blood count and packed cell volume) and faecal parasitic analysis.

Prior to the experiment, food and water were withheld from the goats 12 and 6 h respectively. The experiment was carried out in the morning. The animals were weighed before each treatment performed for calculation of drug dosages. Two animals were studied at any one time. The animals were unable to see or interact with each other. The Skin over the left jugular vein was prepared aseptically for IV administration. The animals were gently restrained on the special bed (on top of a soft mattress) in a quiet, covered, 5 × 6 m^2^ area and rested for 20 min before first measurement of physiological variables were recorded.

### 2.2 Experimental procedures

The goats were assigned to five IV treatments in a randomised (computer‐generated) crossover Latin square design with a minimum washout period of 8 days between treatments. Treatments were included: control (normal saline, 5 ml); XYL, xylazine (100 μg kg^−1^; Xyla, 2%; Interchemie Werken De Adelaar B.V., Netherlands); DET, detomidine (50 μg kg^−1^; Domosedan, Orion Corporation, Finland); MED, medetomidine (20 μg kg^−1^; DorbeneVet; N‐Vet AB, Sweden) and DEX, dexmedetomidine (5 μg kg^−1^; Dexdomitor; Orion Corporation, Finland). The injection volumes of treatments were the same for each animal by dilution with normal saline to 5 ml. Treatments were administered IV in the left jugular vein (over 20 s) via an 18 gauge needle with the animals standing. All investigators recording measurements were blinded to the treatment assigned.

### 2.3 Sedation scores

Three independent observers (who were unaware of the drug type and dose) assessed the degree of sedation for each animal. The degree of sedation was investigated using a numerical ranking scale of 0–10, as follows: 0, no sedation; 1, standing, conscious, decrease head and ear movements; 2, standing, mild head drop; 3, standing, moderate head drop; 4, standing, severe head drop, incoordination; 5, standing, severe head drop, severe incoordination; 6, sternal recumbency, head up; 7, sternal recumbency, head down; 8, lateral recumbency, occasional attempts to attain sternal recumbency; 9, lateral recumbency, uncoordinated movements and 10, lateral recumbency, no movements (Borges et al., 2016; De Carvalho et al., 2016; Kästner et al., 2003). The final sedation score for each animal was considered the majority score which the observers gave to each animal. Sedation scores were recorded before other measurements at the following times: baseline (before drug administration, time 0) and at 5, 10, 15, 30, 45, 60, 90 and 120 min, resulting in nine time points for each animal.

### 2.4 Physiological variables

Physiological variables including HR, RR, RT, CRT and ruminal motility were recorded at the same time points as the sedation was recorded. HR and RR was assessed using veterinary stethoscope (Classic SE Littmann; 3 M, MN, USA) on the left 4th and 6th intercostal space, respectively, for 1 min. Ruminal motility was recorded by auscultation with a stethoscope placed on the left flank. Number of audible rumen contractions within 2 min was counted. CRT was measured by finger pressing on the labial surface of gingiva in the incisor region. A digital thermometer (FT09; Beurer GmbH, Germany) was used to performed RT. Thermometer was 4–5 cm deep in touch with rectal mucosa for at least 2 min (Constable et al., 2017).

### 2.5 Statistical analysis

A prospective power calculation (G^*^Power Version 3.1.9.2) conducted on the basis of information reported elsewhere (Borges et al., 2016; De Carvalho et al., 2016) determined that a total of ten animals were required (α of 0.05 and β of 0.2) to detect a 20% difference between treatments. Data were analysed using SPSS software version 20 (SPSS for Windows, SPSS Inc, Chicago, Illinois, USA). Before any statistical analysis, distribution of data was performed for normality using the Kolmogorov–Smirnov test and normality of data distribution was verified. Sedation scores and physiological variables were expressed as median (range) and mean ± standard deviation, respectively. Sedation scores were compared at each time using nonparametric (Kruskal–Wallis and Mann–Whitney *U*) tests. The two‐relate‐samples test with Wilcoxon test type (in nonparametric method) was applied to compare sedation scores at different times from baseline. One‐way analysis of variance (ANOVA) with Tukey's post hoc test was used to compare mean values of physiological variables at similar times between different treatments. The paired sample *t*‐test was applied to compare physiological variables at different times from baseline. The inter‐rater agreement between the investigators (for each treatment) was performed using Cohen's kappa (*k*) coefficient. The correlations were ranged (very good, *k* = 0.81–1.00; good, *k* = 0.61–0.80; moderate, *k* = 0.41–0.60; fair, *k* = 0.21–0.40 and poor, *k* < 0.20) based on the model set by Altman (1990). A *p* value of less than 0.05 was considered significant.

## 3 RESULTS

All the goats had recovered by 3 h based on behavioural sings such as standing, head up, head and ear movement, consciousness and responsiveness. The inter‐rater agreement among the observers was very good (*k* = 0.92; *p* < 0.05). Different physiological variables are demonstrated in Table 1. All animals in control showed no sedation at any time point. Animals in α_2_‐adrenergic agonist treatments were sedated at 5–60 min (Table 1). Just one animal in each α_2_‐adrenergic agonist treatment achieved sedation score 1 at 90 and 120 min. Sedation was higher in α_2_‐adrenergic agonist treatments than in control at 5–60 min after drug administration (*p* < 0.05). There were no significant differences among α_2_‐adrenergic agonist treatments at 5–60 min in sedation scores.

**TABLE 1:** Comparison of xylazine, detomidine, medetomidine and dexmedetomidine on sedation scores [median (range)] and physiological variables (mean ± standard deviation) in ten goats. Goats were assigned to five intravenous treatments in a crossover Latin square, randomised (computer‐generated) and blind study: XYL, xylazine (100 μg kg^−1^); DET, detomidine (50 μg kg^−1^); MED, medetomidine (20 μg kg^−1^); DEX, dexmedetomidine (5 μg kg^−1^) and control, normal saline (5 ml)

		**Time (min)**								
**Variables**	**Treatments**	**Baseline**	**5**	**10**	**15**	**30**	**45**	**60**	**90**	**120**
Sedation score (0–10)	Control	0 (0–0)	0 (0–0)	0 (0–0)	0 (0–0)	0 (0–0)	0 (0–0)	0 (0–0)	0 (0–0)	0 (0–0)
	XYL	0 (0–0)	3 (1–4)^[Link]^, ^[Link]^	5 (3–5)^[Link]^, ^[Link]^	6 (4–7)^[Link]^, ^[Link]^	7 (5–9)^[Link]^, ^[Link]^	5 (4–7)^[Link]^, ^[Link]^	4 (4–6)^[Link]^, ^[Link]^	1 (0–1)	0 (0–1)
	DET	0 (0–0)	2 (1–5)^[Link]^, ^[Link]^	3 (3–6)^[Link]^, ^[Link]^	4 (4–7)^[Link]^, ^[Link]^	7 (6–9)^[Link]^, ^[Link]^	6 (5–7)^[Link]^, ^[Link]^	5 (3–7)^[Link]^, ^[Link]^	1 (0–1)	0 (0–1)
	MED	0 (0–0)	2 (2–4)^[Link]^, ^[Link]^	3 (3–6)^[Link]^, ^[Link]^	4 (4–7)^[Link]^, ^[Link]^	6 (6–9)^[Link]^, ^[Link]^	4 (4–7)^[Link]^, ^[Link]^	4 (3–6)^[Link]^, ^[Link]^	0 (0–1)	0 (0–1)
	DEX	0 (0–0)	4 (1–4)^[Link]^, ^[Link]^	5 (3–6)^[Link]^, ^[Link]^	5 (4–7)^[Link]^, ^[Link]^	8 (7–9)^[Link]^, ^[Link]^	7 (5–8)^[Link]^, ^[Link]^	6 (5–8)^[Link]^, ^[Link]^	1 (0–1)	0 (0–1)
HR (beats min^−1^)	Control	130 ± 7	129 ± 6	129 ± 5	128 ± 5	127 ± 4	125 ± 4	125 ± 4	128 ± 3	128 ± 5
	XYL	132 ± 11	106 ± 10^[Link]^, ^[Link]^	98 ± 10^[Link]^, ^[Link]^	99 ± 10^[Link]^, ^[Link]^	100 ± 11^[Link]^, ^[Link]^	102 ± 11^[Link]^, ^[Link]^	104 ± 11^[Link]^, ^[Link]^	117 ± 8^[Link]^, ^[Link]^	130 ± 10
	DET	130 ± 12	105 ± 11^[Link]^, ^[Link]^	96 ± 11^[Link]^, ^[Link]^	97 ± 10^[Link]^, ^[Link]^	99 ± 9^[Link]^, ^[Link]^	101 ± 8^[Link]^, ^[Link]^	103 ± 9^[Link]^, ^[Link]^	115 ± 7^[Link]^, ^[Link]^	127 ± 8
	MED	129 ± 5	102 ± 6^[Link]^, ^[Link]^	93 ± 5^[Link]^, ^[Link]^	96 ± 6^[Link]^, ^[Link]^	98 ± 5^[Link]^, ^[Link]^	101 ± 4^[Link]^, ^[Link]^	102 ± 3^[Link]^, ^[Link]^	114 ± 4^[Link]^, ^[Link]^	128 ± 4
	DEX	129 ± 10	103 ± 8^[Link]^, ^[Link]^	96 ± 7^[Link]^, ^[Link]^	98 ± 7^[Link]^, ^[Link]^	100 ± 6^[Link]^, ^[Link]^	103 ± 5^[Link]^, ^[Link]^	104 ± 5^[Link]^, ^[Link]^	115 ± 5^[Link]^, ^[Link]^	126 ± 6
RR (breaths min^−1^)	Control	36 ± 2	37 ± 2	36 ± 1	36 ± 1	36 ± 2	37 ± 2	37 ± 2	37 ± 2	37 ± 2
	XYL	38 ± 2	37 ± 2	35 ± 2	35 ± 2	32 ± 3^[Link]^, ^[Link]^	32 ± 3^[Link]^, ^[Link]^	32 ± 3^[Link]^, ^[Link]^	34 ± 3^[Link]^, ^[Link]^	37 ± 1
	DET	35 ± 3	35 ± 2	34 ± 2	34 ± 2	32 ± 2^[Link]^, ^[Link]^	32 ± 2^[Link]^, ^[Link]^	32 ± 1^[Link]^, ^[Link]^	34 ± 3^[Link]^, ^[Link]^	35 ± 2
	MED	36 ± 3	36 ± 2	35 ± 2	34 ± 1	32 ± 2^[Link]^, ^[Link]^	32 ± 2^[Link]^, ^[Link]^	32 ± 1^[Link]^, ^[Link]^	34 ± 3^[Link]^, ^[Link]^	35 ± 2
	DEX	36 ± 3	35 ± 3	34 ± 2	34 ± 2	32 ± 2^[Link]^, ^[Link]^	31 ± 2^[Link]^, ^[Link]^	32 ± 1^[Link]^, ^[Link]^	34 ± 4^[Link]^, ^[Link]^	35 ± 3
RT (°C)	Control	38.7 ± 0.3	38.7 ± 0.2	38.7 ± 0.1	38.7 ± 0.1	38.7 ± 0.1	38.6 ± 0.1	38.7 ± 0.1	38.7 ± 0.1	38.8 ± 0.1
	XYL	38.6 ± 0.1	38.6 ± 0.1	38.6 ± 0.2	38.8 ± 0.2	38.8 ± 0.2	38.6 ± 0.3	38.7 ± 0.2	38.5 ± 0.3	38.8 ± 0.2
	DET	38.7 ± 0.1	38.7 ± 0.2	38.6 ± 0.1	38.6 ± 0.3	38.8 ± 0.2	38.7 ± 0.3	38.6 ± 0.3	38.7 ± 0.2	38.6 ± 0.2
	MED	38.7 ± 0.1	38.8 ± 0.1	38.7 ± 0.1	38.6 ± 0.2	38.6 ± 0.1	38.8 ± 0.2	38.6 ± 0.3	38.7 ± 0.2	38.8 ± 0.2
	DEX	38.7 ± 0.1	38.7 ± 0.1	38.7 ± 0.1	38.7 ± 0.3	38.6 ± 0.2	38.5 ± 0.2	38.8 ± 0.2	38.8 ± 0.3	38.5 ± 0.3
Ruminal motility (Contractions min^−1^)	Control	2	2	2	2	2	2	2	2	2
	XYL	2	1^[Link]^, ^[Link]^	0^[Link]^, ^[Link]^	0^[Link]^, ^[Link]^	0^[Link]^, ^[Link]^	0^[Link]^, ^[Link]^	0^[Link]^, ^[Link]^	1^[Link]^, ^[Link]^	1
	DET	2	1^[Link]^, ^[Link]^	0^[Link]^, ^[Link]^	0^[Link]^, ^[Link]^	0^[Link]^, ^[Link]^	0^[Link]^, ^[Link]^	0^[Link]^, ^[Link]^	1^[Link]^, ^[Link]^	1
	MED	2	1^[Link]^, ^[Link]^	0^[Link]^, ^[Link]^	0^[Link]^, ^[Link]^	0^[Link]^, ^[Link]^	0^[Link]^, ^[Link]^	0^[Link]^, ^[Link]^	1^[Link]^, ^[Link]^	1
	DEX	2	1^[Link]^, ^[Link]^	0^[Link]^, ^[Link]^	0^[Link]^, ^[Link]^	0^[Link]^, ^[Link]^	0^[Link]^, ^[Link]^	0^[Link]^, ^[Link]^	1^[Link]^, ^[Link]^	1

HR, heart rate; RR, respiratory rate; RT, rectal temperature.

*Significantly different from control treatment at the same time point (*p* < 0.05).

†Significantly different from baseline value within the same treatment (*p* < 0.05).

The degree of sedation was assessed using a numerical rating scale of 0–10, as follows: 0, no sedation; 1, standing, conscious, decrease head and ear movements; 2, standing, mild head drop; 3, standing, moderate head drop; 4, standing, severe head drop, incoordination; 5, standing, severe head drop, severe incoordination; 6, sternal recumbency, head up; 7, sternal recumbency, head down; 8, lateral recumbency, occasional attempts to attain sternal recumbency; 9, lateral recumbency, uncoordinated movements; and 10, lateral recumbency, no movements (Borges et al., 2016; De Carvalho et al., 2016; Kästner et al., 2003).

HR significantly decreased from baseline at 5–90 min after α_2_‐adrenergic agonists’ administration. HR was significantly lower in α_2_‐adrenergic agonist treatments than control at 5–90 min after drug administration (*p* < 0.05). A significant decrease from baseline in RR was detected between 30–90 min after administration of α_2_‐adrenergic agonists. Compared to control, RR was significantly lower in α_2_‐adrenergic agonist treatments at 30–90 min after drug administration. Ruminal motility was decreased in α_2_‐adrenergic agonist treatments at 5, 90 and 120 min and absent at 10–60 min. Compared to control, ruminal motility was significantly lower in α_2_‐adrenergic agonist treatments at 5–120 min after drug administration. There were no significant differences in HR, RR and ruminal motility among α_2_‐adrenergic agonists at all time points. RT was unchanged in any treatment for 120 min. CRT was less than 2 s at all time points following each treatment.

## 4 DISCUSSION

α_2_‐adrenergic agonists are used for sedation in small ruminants (Kästner, 2006). These drugs bind to α_2_‐agonist receptors in the brain and spinal cord (Kästner et al., 2003). The xylazine and medetomidine doses used in the present study were determined based on other studies in sheep (De Carvalho et al., 2016; Kästner, 2006). Medetomidine at 20 μg kg^−1^ is recommended in goats by Carroll et al. (2005). The dexmedetomidine dose used in the present study was determined based on other study in sheep (Borges et al., 2016). It would not regard that the doses used in this study as equipotent. Medetomidine contains 50% active dexmedetomidine, so 20 μg kg^−1^ medetomidine should be twice as potent as 5 μg kg^−1^ dexmedetomidine. In this experiment, dexmedetomidine (at 5 μg kg^−1^) appeared to be at least as potent as medetomidine (at 20 μg kg^−1^). According to the results, the hypothesis of this study was disproved and there is no significant differences were detected between α_2_‐adrenergic agonists in goats. It may be due to the doses and pharmacological characteristics of α_2_‐adrenergic agonists used in this study (Celly et al., 2003; Kästner, 2006).

α_2_‐adrenergic agonists suppress the vasomotor centre in brainstem in the central nervous system (CNS). Sedation is associated with decrease in sympathetic outflow from the CNS (Pawde et al., 1996). In this study, animals in α_2_‐adrenergic agonist treatments were sedated at 5–60 min. In a study carried out in sheep, treatment with 30 μg kg^−1^ detomidine, 10 μg kg^−1^ medetomidine or 100 μg kg^−1^ xylazine resulted in sedation lasting 60 min (Celly et al., 2003). Administration of 100 μg kg^−1^ xylazine (Borges et al., 2016) or 5 μg kg^−1^ dexmedetomidine (De Carvalho et al., 2016) in sheep was reported to produce sedation lasting 90 min. It should be noted that sex, age, species and breed, rout of administration and also other factors including environmental variables may affect the sedation in farm practice (Kästner et al., 2003). Good sedation is important to perform clinical procedures (surgical and non‐surgical) efficiently and properly. Veterinarians can do a better job when the animal is not moving, struggling and/or vocalising (Seddighi & Doherty, 2016). In this study, no significant differences were detected among α_2_‐adrenergic agonists in sedation scores. However in the country the experiment took place, xylazine is readily available and very inexpensive, while detomidine and dexmedetomidine are much more expensive. Medetomidine is not readily available anymore.

All α_2_‐adrenergic agonists in this study a significant reduction in HR 5–90 min after drug administration. Pawde et al. (1996) reported to produce bradycardia for 90 min by 15 μg kg^−1^ medetomidine in goats. Administration of 5 μg kg^−1^ dexmedetomidine was reported to produce bradycardia for 120 min in sheep (Borges et al., 2016). Treatment with 2 μg kg^−1^ dexmedetomidine (Kutter et al., 2006) or 100 μg kg^−1^ xylazine (De Carvalho et al., 2016) resulted in bradycardia lasting 120 min in sheep, whereas administration of 100 μg kg^−1^ xylazine, 30 μg kg^−1^ detomidine or 10 μg kg^−1^ medetomidine was reported to produce bradycardia lasting 90 min (Celly et al., 2003).

α_2_‐adrenergic agonists cause a significant reduction in RR between 30 and 90 min after drug administration. RR was decreased for 40 min after IV administration of xylazine (100 μg kg^−1^) and returning to normal at 105 min in sheep (De Carvalho et al., 2016). Bryant et al. (1998) reported to produce bradypnea for 5–60 min by 5 μg kg^−1^ medetomidine in sheep. Celly et al. (2003) reported to produce bradypnea for 30–60 min by 100 μg kg^−1^ xylazine, 50 μg kg^−1^ detomidine or 10 μg kg^−1^ medetomidine in sheep. Treatment with 5 μg kg^−1^ dexmedetomidine or 200 μg kg^−1^ xylazine resulted in bradypnea for 5–60 min in dromedary calves (Samimi et al., 2020).

Ruminal motility was decreased in α_2_‐adrenergic agonist treatments at 5–120 min. Similarly, ruminal motility was decreased for about 120 min after IV xylazine (200 μg kg^−1^), medetomidine (20 μg kg^−1^) or dexmedetomidine (5 μg kg^−1^) in dromedary calves (Samimi et al., 2019; Samimi et al., 2020). Decreased in physiological variables (HR, RR and ruminal motility) following α_2_‐adrenergic agonists have been reported in sheep and goats (Kästner et al., 2003; Kästner et al., 2007; Kutter et al., 2006) and camels (Samimi et al., 2019). By affecting the hormonal and nervous systems, α_2_‐adrenergic agonists reduce HR, RR and gastrointestinal motility (Celly et al., 2003; Kästner, 2006; Kutter et al., 2006).

No significant differences were observed in RT and CRT at different time points in each treatment in this study. Similarly, RT and CRT were not change for about 120 min after IV administration of medetomidine (15 μg kg^−1^) in goats (Pawde et al., 1996) and after IV xylazine (200 μg kg^−1^), medetomidine (20 μg kg^−1^) or dexmedetomidine (5 μg kg^−1^) in dromedary calves (Samimi et al., 2019; Samimi et al., 2020).

Assessment of only HR and RR are inadequate to describe the cardiorespiratory effects of these drugs. Evaluation of stroke volume, blood pressure, pulmonary arterial pressure, pH and partial pressures of arterial oxygen and carbon dioxide would describe the effects of these drugs.

## 5 CONCLUSION

α_2_‐adrenergic agonists have sedative effects on goats. The duration of sedation was up to 60 min after IV administration of xylazine (100 μg kg^−1^), detomidine (50 μg kg^−1^), medetomidine (20 μg kg^−1^) and dexmedetomidine (5 μg kg^−1^) in goats. No significant differences were detected between xylazine, detomidine, medetomidine and dexmedetomidine in goats. More investigations with evaluation of cardiorespiratory effects are recommended.

## CONFLICT OF INTEREST

The authors declare that they have no competing interests.

## ETHICAL APPROVAL

All ethical considerations including utilising animals were considered cautiously. The trial convention was affirmed by the animal welfare committee (which was covered IACUC approval) of the Faculty of Veterinary Medicine, Shahid Bahonar University of Kerman, Kerman, Iran (institutional approval number IR.UK.VETMED.REC.1399.014).

All applicable international, national and/or institutional guidelines for the care and use of animals were followed.

## AUTHOR CONTRIBUTIONS

A. Nahvi: data curation; funding acquisition; investigation; methodology; visualisation; writing‐original draft; formal analysis; project administration. M.M. Molaei: conceptualisation; funding acquisition; investigation; methodology; project administration; supervision; validation; visualisation. A.S. Samimi: conceptualisation; data curation; formal analysis; project administration; resources; writing‐review & editing; software; supervision; visualisation; writing‐original draft. O. Azari: conceptualisation; funding acquisition; investigation; methodology; project administration; resources; supervision; validation; visualisation. H. Mashayekhi: data curation; funding acquisition; investigation; project administration; software; writing‐original draft. F. Ebrahimzadeh: formal analysis; funding acquisition; investigation; methodology; writing – original draft. The authors approved the final version of this manuscript.

## Data Availability

The data that support the findings of this study are available from the corresponding author upon reasonable request.
